# Gene-metabolite network analysis in different nonalcoholic fatty liver disease phenotypes

**DOI:** 10.1038/emm.2016.123

**Published:** 2017-01-13

**Authors:** Xiao-Lin Liu, Ya-Nan Ming, Jing-Yi Zhang, Xiao-Yu Chen, Min-De Zeng, Yi-Min Mao

**Affiliations:** 1 Division of Gastroenterology and Hepatology, Renji Hospital, School of Medicine, Shanghai Jiao Tong University, Shanghai Institute of Digestive Disease, Shanghai, China

## Abstract

We sought to identify common key regulators and build a gene-metabolite network in different nonalcoholic fatty liver disease (NAFLD) phenotypes. We used a high-fat diet (HFD), a methionine-choline-deficient diet (MCDD) and streptozocin (STZ) to establish nonalcoholic fatty liver (NAFL), nonalcoholic steatohepatitis (NASH) and NAFL+type 2 diabetes mellitus (T2DM) in rat models, respectively. Transcriptomics and metabolomics analyses were performed in rat livers and serum. A functional network-based regulation model was constructed using Cytoscape with information derived from transcriptomics and metabolomics. The results revealed that 96 genes, 17 liver metabolites and 4 serum metabolites consistently changed in different NAFLD phenotypes (>2-fold, *P*<0.05). Gene-metabolite network analysis identified ccl2 and jun as hubs with the largest connections to other genes, which were mainly involved in tumor necrosis factor, P53, nuclear factor-kappa B, chemokine, peroxisome proliferator activated receptor and Toll-like receptor signaling pathways. The specifically regulated genes and metabolites in different NAFLD phenotypes constructed their own networks, which were mainly involved in the lipid and fatty acid metabolism in HFD models, the inflammatory and immune response in MCDD models, and the AMPK signaling pathway and response to insulin in HFD+STZ models. Our study identified networks showing the general and specific characteristics in different NAFLD phenotypes, complementing the genetic and metabolic features in NAFLD with hepatic and extra-hepatic manifestations.

## Introduction

Nonalcoholic fatty liver disease (NAFLD) encompasses a spectrum of diseases ranging from nonalcoholic fatty liver (NAFL) to nonalcoholic steatohepatitis (NASH).^1^ Although NAFLD is benign and can generally be reversed by diet and exercise, it has the potential to generate quite serious medical consequences such as cirrhosis, hepatocellular carcinoma and earlier onset of type 2 diabetes mellitus (T2DM).^2^ Consequently, NAFLD is increasingly recognized as a low-grade systemic inflammatory state accompanied by hepatic and extra-hepatic manifestations.^3^

Recent studies have shown that cardiovascular disease (30%) and extra-hepatic malignancy (28%) are the most common causes of death in patients with NAFLD rather than liver disease (19%).^4^ Therefore, more attention should be paid to the cardiovascular metabolic risk in NAFLD. Transcriptomics is an emerging high-throughput screening technique that enables detection of small changes in messenger RNA expression sensitively through microarray analysis, whereas metabolomics have the ability to quantify and characterize small metabolites in tissues and biofluids. The combination of transcriptomics and metabolomics is often preferred as a powerful tool in the study of multisystem diseases such as NAFLD.^5^ Several studies have identified regulated genes and pathways in NAFLD, including stearoyl-coenzyme A desaturase 1 (Scd1), sterol regulatory element binding factor 1c (Srebf1c) and the disturbances of phospholipid and bile acid metabolism.^6, 7^ However, these studies focused exclusively on one NAFLD phenotype (that is, NAFL or NASH) without taking extra-hepatic manifestations of NAFLD into account.

Given the numerous complex and influencing factors in the human system, we used rat models to mimic disease manifestations in NAFLD patients in controlled genetic and environmental conditions. In this study, high-fat diet (HFD), methionine-choline-deficient diet (MCDD) and HFD+STZ rat models were used to represent simple fatty liver, fatty liver presenting with severe hepatic manifestations and fatty liver presenting with severe metabolic manifestations, respectively.^8, 9, 10^ We applied liver-transcriptomics (that is, microarray analysis), and liver and serological-metabolomics (that is, liquid chromatography-mass spectrometry, LC-MS) to characterize the commonly and specifically regulated genes and metabolomics in different NAFLD phenotypes, thereby attempting to determine key regulators and adaptive pathways in this disease.

## Materials and methods

### Animals and treatments

Forty male Sprague–Dawley rats (6 weeks old) were housed in controlled conditions (24±2 °C and a 12 h light/dark cycle) with free access to food and water. They were randomized into four experimental groups: the control group received a normal diet; the second group was fed a HFD(60 kcal%, Research Diets, New Brunswick, NJ, USA) to establish the NAFL model; the third group received a MCDD(Research Diets, USA) to establish the NASH model; and the fourth group was fed a HFD with a single intraperitoneal injection of streptozotocin (STZ; Sigma, St Louis, MO, USA; 25 mg kg^−1^ in 0.1 M citrate buffer) at the end of 11 weeks to establish the NAFL+T2DM model. A hepatic steatosis observed in the liver biopsy and an NAFLD activity score (NAS) <5 indicated the establishment of the NAFL model, whereas significant hepatic steatosis with hepatocellular injury and NAS ⩾5 indicated the presence of NASH.^11^ The criteria for successfully establishing the T2DM model is fasting blood glucose ⩾11.1 mmol l^−1^ by two consecutive measurements.^12^ This experiment followed the National Research Council's Guide for the Care and Use of Laboratory Animals and was approved by the Institutional Animal Care and Use Committee of SLAC (IACUC). All our animal experiments were performed in accordance with the IACUC Guide for Care and Use of Laboratory Animals.

### Serum and tissue sampling

All rats were killed by decapitation after fasting overnight. Blood samples were collected from the retinal venous plexus. Parts of the liver were either fixed in 4% formaldehyde overnight for histological examination or snap-frozen for later analysis.

### Clinical chemistry and histological analysis

Serum alanine aminotransferase, aspartate aminotransferase, total bilirubin, triglyceride, high-density lipoprotein, low-density lipoprotein and fasting blood glucose were measured using an automatic biochemical analyzer (Siemens Advia 1800: Siemens Healthcare Diagnostics, Tarrytown, NY, USA), and fasting insulin was measured using a gamma radioimmunoassay counter (SN-697, Hesuo Rihuan, Shanghai, China). Paraffinized and frozen liver sections were stained with hematoxylin and eosin (H&E) and oil red O, respectively. Histopathological features of steatosis, lobular inflammation and hepatocellular ballooning were scored according to the Non-alcoholic Steatohepatitis Clinical Research Network standard, and NAS was the sum of the scores in each component.

### Gene expression profiling and data analysis

Total RNA from livers of 12 rats (three rats per group) was extracted using Trizol reagent (Takara, Shiga, Japan). Microarray analysis was performed with the rat OneArray (Phalanx Biotech Group, Hsinchu, Taiwan) that contained 24 358 genes according to the manufacturer's instructions.^13^ After probe filtering, the background value, signal intensity, signal-to-noise ratio and probe identification were collected as raw data. The raw data were normalized by median scaling of the technical repeats, and the mean was defined as the normalized probe signal. Standard selection criteria for significant differentially expressed genes were defined as log_2_ |Fold change| ⩾1, and adjusted *P*-value <0.05. Principal component analysis and clustering analysis were performed on the differentially expressed genes.^14^ The gene expression information in this experiment was submitted to the Gene Expression Omnibus database with the registration no. GSE65220.

### Validation of microarray data using real-time quantitative PCR

To validate findings of microarray analysis and quantify other genes of interest, representative gene subsets that were involved in regulated signaling pathways, including tumor necrosis factor (TNF), P53, nuclear factor-kappa B (NF-κB), chemokine, peroxisome proliferator activated receptor (PPAR) and Toll-like receptor (TLR) signaling pathways, were analyzed using real-time quantitative polymerase chain reaction (qRT-PCR) assay. Values were normalized to those of the 18s rRNA sub-unit and are calculated based on the comparative 2^ΔΔCt^ method.

### Ultra-performance liquid chromatography/mass spectrometric analysis

A global metabolite profile by ultra-performance liquid chromatography-mass spectrometry was performed to study metabolic differences among different groups. Tissue from each liver was homogenized and centrifuged for 10 min at 12 000 *g* and 4 °C. After centrifugation, 80 μl supernatant was separated for later analysis. Each serum sample was mixed with methanol and incubated, centrifuged for 10 minute at 12 000 *g* and 4 °C. Then, 200 μl supernatant was transferred into analytical vials for later analysis. Ultra-performance liquid chromatography-mass spectrometry was performed on a LC-Q/TOF-MS platform (Agilent, Santa Clara, CA, USA, 1290 Infinity LC, 6530 UHD and Accurate-Mass Q-TOF/MS). The data were visualized by principal component analysis and orthogonal partial least-squares discriminant analysis models.^15^ The differentially expressed metabolites were selected according to the variable importance in the projection of the orthogonal partial least-squares model combined with the *P*-value of Student's *t*-test, which was identified by searching an online database (http://metlin.scripps.edu/).

### Combined analysis of liver transcriptomics and liver and serum metabolomics

Official gene names and unique collision-induced dissociation (CID) for differentially expressed genes and metabolites were used to perform pathway enrichment analysis in the Kyoto Encyclopedia of Genes and Genomes (KEGG), human metabolome database (HMDB) and PubMed database. KEGG is a manually curated pathway database. According to the KEGG database, pathways are clustered into sub-categories, including Metabolism, Genetic Information Processing, Environmental Information Processing, Cellular Processes, Organismal Systems and Human Diseases. Enrichment analysis of KEGG pathways was performed to test whether a KEGG term is statistically enriched for the given set of genes. The hypergeometric test is the most common statistical method for enrichment analysis: 
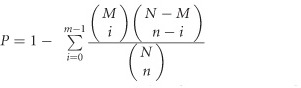
 where *N* is the number of all genes of the specific organism that were annotated in KEGG (Background Genes); *n* is the number of query genes annotated to the KEGG Term; *M* is the number of all genes that are annotated to certain KEGG terms (Pop Hit); and *m* is the number of query genes annotated to certain KEGG terms (count). The enriched pathways were used for model construction. The protein–protein and protein–compound interaction analysis was performed with differentially expressed genes and related enzymes of differentially expressed metabolites via the STRING database (version 9.1).^16^ Cytoscape software (San Diego, CA, USA, version 3.1.1) was used to construct a functional network-based regulatory model.^17^

### Statistical analysis

The data were analyzed using SPSS version 16.0 (Chicago, IL, USA) statistical software. Quantitative data were presented as the mean±s.e.m. Statistical comparisons among groups were made by one-way analysis of variance with a Newman–Keuls *post hoc* test. Differences were considered statistically significant at an alpha value of *P*<0.05.

## Results

### The characteristics of HFD, MCDD and HFD+STZ rat models

**Figure 1:**
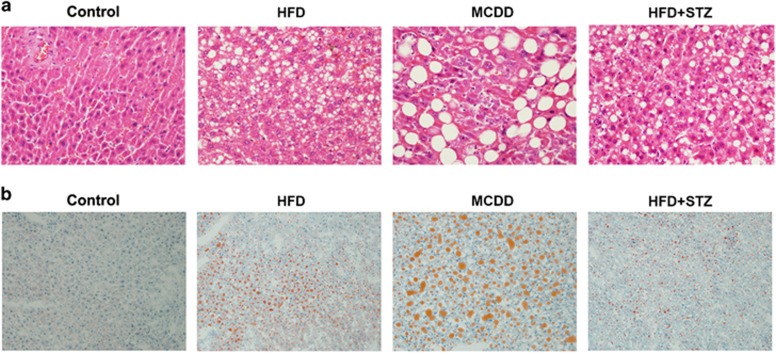
The establishment of HFD, MCDD and HFD+STZ rat models. (**a**) H&E staining (× 200 magnification) and (**b**) Oil-Red-O staining (× 200 magnification) of the liver in different groups.

After 12 weeks of treatment, HFD and HFD+STZ rats exhibited increased body weight, but MCDD rats exhibited decreased body weight. The histological examination of the liver showed that HFD and HFD+STZ groups exhibited diffusely mixed hepatic steatosis, and their NAS ranged from 2 to 4 and 3 to 5, respectively. Significant macrovesicular steatosis accompanied by lobular inflammation was present in the NASH group, and its NAS ranged from 5 to 7 (Figure 1). After STZ injection, all HFD+STZ rat models showed an fasting blood glucose >11.1 mmol l^−1^ in two consecutive measurements. The NASH group had 10- and 5-fold increased serum alanine aminotransferase and aspartate aminotransferase levels, and both HFD and HFD+STZ groups had a 4- and 6-fold increased HOMA-IR levels, respectively (*P*<0.05; Table 1). These results indicated that HFD, MCDD and HFD+STZ rat models were all successfully established, with more severe liver injury found in the MCDD group and advanced metabolic disorders noted in the HFD+STZ group.

### Analysis of hepatic genes commonly altered in different NAFLD phenotypes

**Figure 2:**
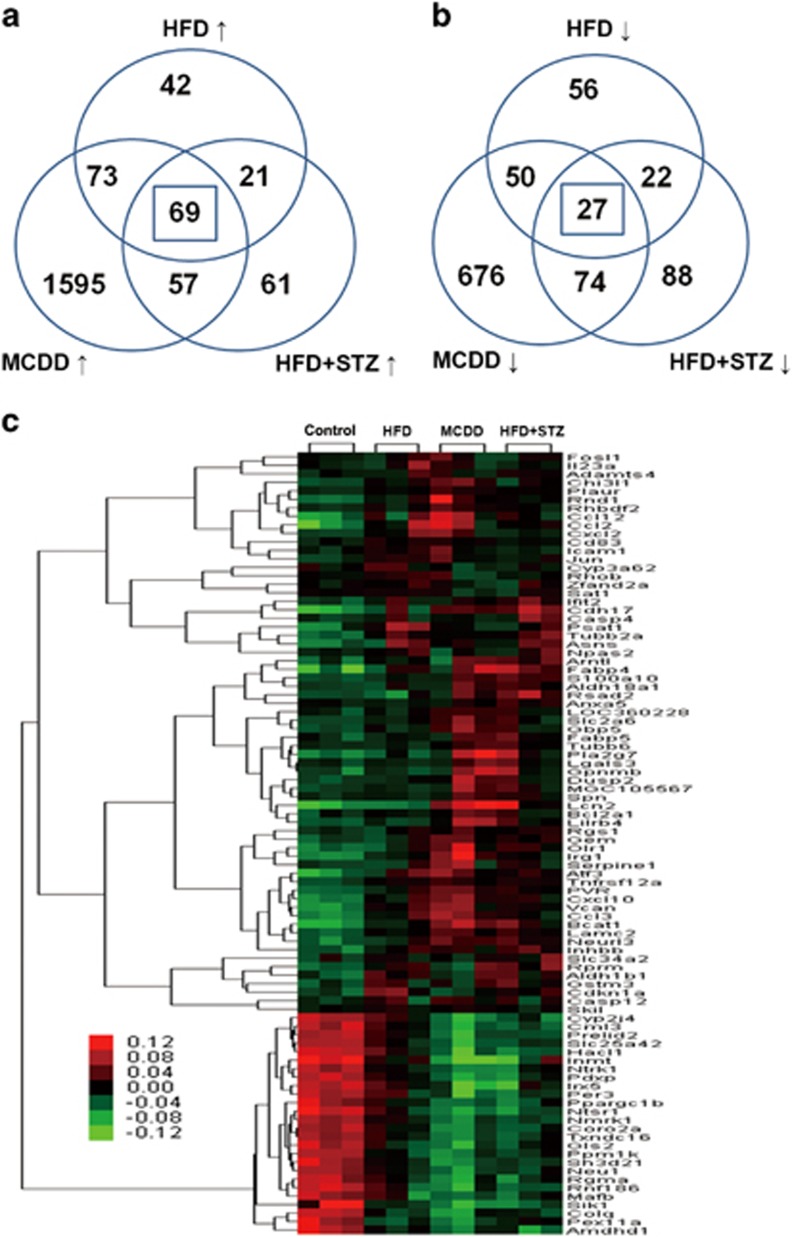
Microarray analysis of the hepatic genes in different phenotypes of NAFLD. (**a**) Venn diagram illustrating the distinct and overlapping genes that were upregulated. (**b**) Venn diagram illustrating the distinct and overlapping genes that were downregulated. (**c**) The heat-map of 96 genes that were expressed in different phenotypes of NAFLD.

According to our defined filtering criteria, we identified 205, 1794 and 208 genes that were upregulated and 155, 827 and 211 genes that were downregulated in HFD, MCDD and HFD+STZ groups, respectively, compared with the control group (*P*<0.05; Supplementary Tables S1–S3). In total, 69 upregulated and 27 downregulated genes exhibited the same regulation among different NAFLD phenotypes (Figures 2a and b). The heat map that was produced by unsupervised hierarchical clustering of these 96 commonly changed genes revealed that samples clustered into the control and experiment groups (that is, HFD, MCDD and HFD+STZ groups; Figure 2c). Pathway enrichment analysis for genes that were commonly regulated among HFD, MCDD and HFD+STZ rat livers showed that TNF, P53, NF-κB and chemokine signaling pathways were among the most significantly regulated pathways (*P*<0.05; Table 2). Moreover, differentially regulated genes that were involved in PPAR and TLR signaling pathways were also included in the model construction given that these signaling pathways have important roles in the development of NAFLD.

### Analysis of hepatic and serological metabolites commonly changed in different NAFLD phenotypes

**Figure 3:**
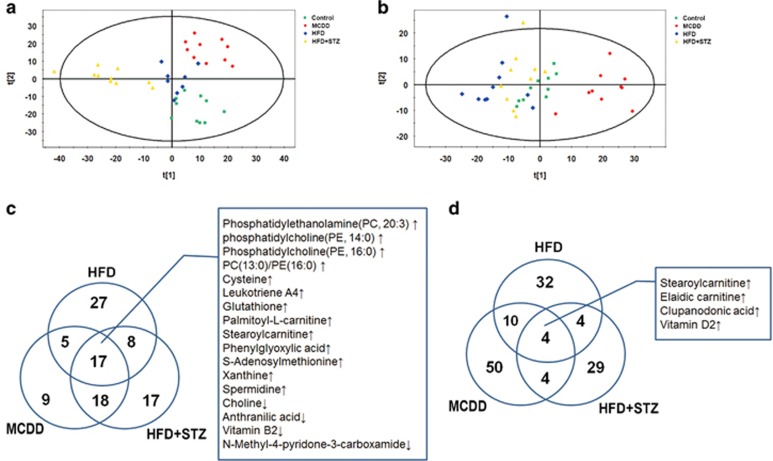
The metabolomic analysis of liver tissue and serum samples in different phenotypes of NAFLD. The score plot of the principal component analysis that was derived from the ultra-performance liquid chromatography-mass spectrometry (UPLC-MS) spectra of liver tissues (**a**) and serum (**b**), indicating discrimination among different groups. Venn diagram illustrates the distinct and overlapping metabolites and lists of metabolites that commonly changed in different phenotypes of NAFLD in liver tissue (**c**) and serum samples (**d**) of the rat model groups.

Ultra-performance liquid chromatography-mass spectrometry spectra were visualized through principal component analysis to determine the actual clusters, wherein the results showed clear metabolic differentiation among control, HFD, MCDD and HFD+STZ rat models both in the liver tissues and serum (Figures 3a and b). According to liver tissue metabolomic analysis, 57, 49 and 60 metabolites were significantly altered in the HFD, MCDD and HFD+STZ groups, respectively (Supplementary Table S4; *P*<0.05); in addition, 17 metabolites were commonly altered among the experimental groups (Figure 3c). The serological metabolomic data revealed that 50, 68 and 41 metabolites were changed in the HFD, MCDD and HFD+STZ groups, respectively (Supplementary Table S5; *P*<0.05). There were four metabolites commonly augmented among the three experimental groups (Figure 3d). Liver and serum metabolomics showed that stearoyl-carnitine was the only altered metabolite in both the liver and serum samples derived from the three NAFLD phenotypes. Pathway enrichment analysis of the metabolites that were commonly changed in HFD, MCDD and HFD+STZ groups was performed using the KEGG, the HMDB and PubMed databases. The significantly regulated metabolic pathways included bile secretion, glutathione metabolism, arachidonic acid metabolism, unsaturated fatty acid biosynthesis, mitochondrial beta-oxidation of long chain saturated fatty acids, fatty acid degradation, carnitine/acylcarnitine translocase and autophagy regulation (*P*<0.05; Table 3).

### Combined analysis of liver transcriptomics and liver and serum metabolomics

To identify the key transcription factors and underlying regulatory mechanisms that were associated with the pathogenesis in different phenotypes of NAFLD, we performed a combined analysis of liver transcriptomics and liver and serum metabolomics. This model included modules for signaling pathways, metabolites and genes.

**Figure 4:**
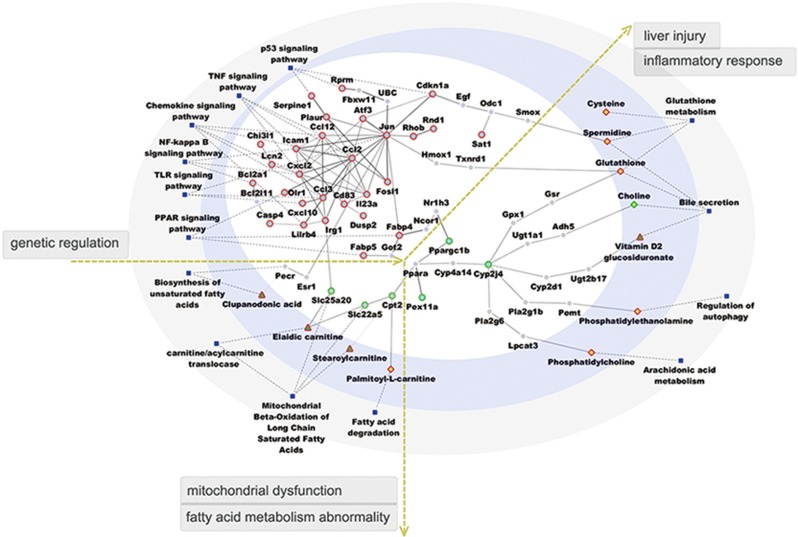
The functional network-based regulation model with genes and metabolites commonly regulated in different NAFLD phenotypes. This model includes modules of genes, metabolites and signaling pathways. Circle nodes represent genes; rhombus and triangle nodes represent metabolites in the liver and serum, respectively; borders of the nodes represent the types of gene and metabolites (that is, those that were upregulated are shown in red, and those that were downregulated are shown in green); genes that were not quantified are presented in gray; and blue filled square nodes indicate signaling pathways. The gene–gene and gene–metabolic interactions are depicted as gray solid line; dashed lines depict the linkage of genes to related signaling pathways.

In the gene-metabolic networks that were associated with the commonness in three phenotypes of NAFLD, the regulated genes were mostly involved in TNF, P53, NF-κB, chemokine, PPAR and TLR signal transduction pathways (Figure 4). Chemokine (C–C Motif) Ligand 2 (Ccl2) and jun were identified as key transcriptional regulators that were relevant to NAFLD progression. In NAFLD, ccl2 affected ccl3 and immunoresponsive 1 homolog (irg1), which may dampen solute carrier family 25, member 20 (slc25a20) and induce an increase in serum elaidic carnitine. Ccl2 also had a regulatory role in jun and fatty acid binding protein 4 (fabp4), which may lead to the downregulation of carnitine palmitoyltransferase 2 (cpt2) and solute carrier family 22 member 5 (slc22a5), causing an increase in stearoyl carnitine and palmitoyl-L-carnitine. In addition, jun-regulated estrogen receptor 1 (esr1) and peroxisomal trans-2-enoyl-CoA reductase (pecr), which led to an increase in serum clupanodonic acid. These metabolic pathways influenced the biosynthesis of unsaturated fatty acids, mitochondrial beta-oxidation of long chain saturated fatty acids, fatty acid degradation and carnitine/acylcarnitine translocase, which collectively indicated mitochondrial dysfunction and abnormalities in fatty acid metabolism. Jun had a regulatory role in cyclin-dependent kinase inhibitor 1A (cdkn1a) and heme oxygenase 1 (hmox1), leading to an increase in glutathione and spermidine production in the liver. In addition, jun affected fabp4 and cytochrome P450, family 2, subfamily J, polypeptide 4 (cyp2j4), inducing an increase in the levels of phosphatidylethanolamine, phosphatidylcholine and glutathione in the liver and vitamin D2 glucosiduronate in the serum and decreases in the levels of choline in the liver. These secreted agents could induce an inflammatory response and liver injury by impairing bile secretion, glutathione metabolism, arachidonic acid metabolism and regulation of autophagy.

**Figure 5:**
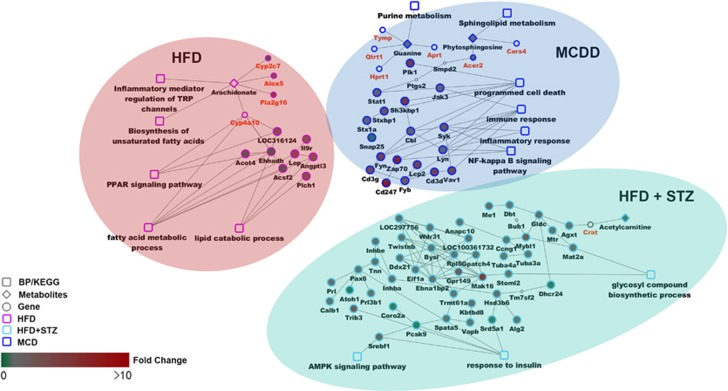
The functional network-based regulation models with genes and metabolites specifically regulated in HFD, MCDD and HFD+STZ rats. This model includes modules of genes, metabolites and signaling pathways.

In the gene-metabolic networks that were constructed with genes and metabolites specifically deregulated in different NAFLD phenotypes, we identified the dysfunction of lipid and fatty acid metabolism and PPAR signaling pathway in HFD rats; the dysfunction of immune and inflammatory response, programmed cell death, and NF-κB signaling pathway in MCDD rats; and the dysfunction of glycosyl compound biosynthetic process, response to insulin and AMPK signaling pathway in HFD+STZ rats (Figure 5).

### Validation of genes involved in regulated signaling and metabolic pathways

**Figure 6:**
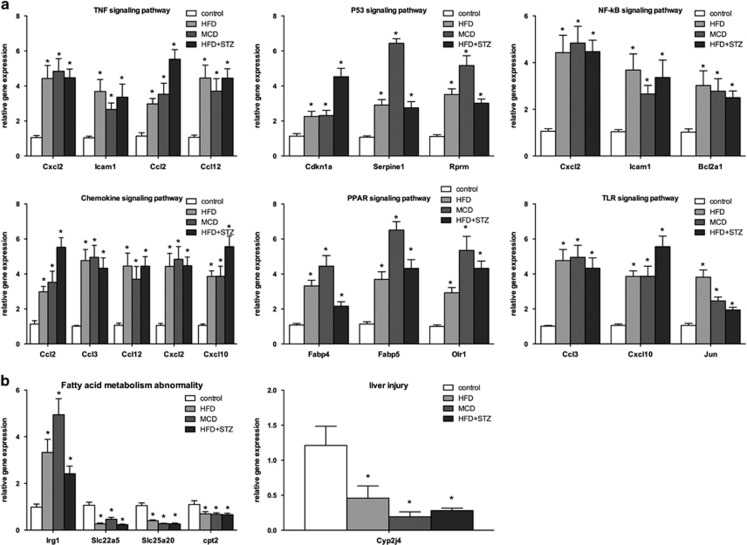
Validation of selected genes involved in genetic and metabolic pathways by qRT-PCR assays. Hepatic mRNA levels of genes associated with TNF, P53, NF-κ, chemokine, PPAR and TLR signaling pathways (**a**), and those genes associated with abnormal fatty acid metabolism and liver injury (**b**) are also shown. The mRNA levels were normalized to those of 18S and subsequently normalized to those of the control group. *P<0.05 compared with the control group.

On the basis of the functional network-based regulation model, we chose representative gene subsets that were directly involved in TNF, P53, NF-κB, chemokine, PPAR and TLR signaling pathways for validation. The mRNA levels of these genes were measured by real-time qRT-PCR. Compared with the control group, the mRNA levels of ccl2, ccl3, ccl12, cxcl2, cxcl10, icam1, cdkn1a, serpine1, rprm, bcl2a1, fabp4, fabp5, olr1 and jun exhibited a two- to six-fold increase in the experimental groups (*P*<0.05; Figure 6a). These results demonstrated the activation of TNF, P53, NF-κB, chemokine, PPAR and TLR signaling pathways in different NAFLD stages. In addition, we examined the expression levels of igr1, slc22a5, slc25a20 and cpt2, which are important genes that regulate fatty acid metabolism. The results showed a two- to five-fold increase in irg1 but a 0.25- to 0.5-fold decrease in slc22a5, slc25a20 and cpt2 in the experimental groups. As cyp2j4 was defined as a key regulator of inflammatory responses and liver injury in different NAFLD phenotypes, we also tested the mRNA expression level of cyp2j4, which showed a significant decrease in all experiment groups (*P*<0.05; Figure 6b).

## Discussion

Our study has focused on the key regulators and adaptive pathways in different NAFLD phenotypes and identified the gene-metabolic networks in NAFLD with severe hepatic manifestations and/or with severe extra-hepatic manifestations.

Liver inflammation is an integral part of NAFLD, which is characterized by upregulation of chemokine, and ccl2 is a prominent example.^18^ Ccl2 has an important role in stimulating TNF-α expression via the NF-κB pathway, and TNF-α subsequently enhances ccl2 secretion to promote exacerbated inflammation.^19^ Our results showed that ccl2 had a regulatory role in ccl12, icam1, ccl3 and cxcl2, which belong to the TNF and NF-κB signaling pathways. NF-κB activation promotes inflammatory cytokine expression and participates in the occurrence and development of insulin resistance,^20^ which is associated with the pathogenesis and progression of NAFLD. Thus, we suggest the hypothesis that ccl2 has an important role in different stages of NAFLD.

In addition, jun is an essential mediator of TLR-induced inflammation and a critical target of Jun N-terminal kinase (JNK) in mediating activation of the p53 signaling pathway.^21, 22^ The TLR signaling pathway is associated with the production of pro-inflammatory cytokines and activation of innate immunity and might also activate Jun N-terminal kinase and NF-κB signaling in the development of NAFLD.^23, 24^ In our experiments, increases in jun, ccl3 and cxcl10 in the TLR-mediated signaling pathway provided supporting evidence for this hypothesis. As NAFLD progresses, hepatocyte apoptosis is induced by the p53 signaling pathway,^25^ which is accompanied by the enhanced expression of cdkn1a, serpine1 and rprm. A relationship exists between jun and fabp4, and the latter is a key regulator in the PPAR signaling pathway. Fabp contributes to atherosclerosis development as it induces insulin resistance and potentiates lipid-induced inflammation,^26^ which are collectively important pathogenic processes that are involved in NAFLD.

Our gene-metabolite network-based regulation model showed that ccl2 and jun regulated each other and triggered activation of numerous signaling pathways. The increased expression of fabp4 and irg1 might inhibit cpt2 and cyp2j4, which collectively could lead to mitochondrial dysfunction and inflammatory responses. The significantly inhibited cpt2 and slc22a5 increases stearoyl carnitine, palmitoyl-L-carnitine and elaidic carnitine levels, which ultimately decrease fatty acid β-oxidation.^27^ Enhanced expression of irg1 may also inhibit the expression of slc25a20, thereby increasing serum levels of elaidic carnitine. The disturbances in this metabolic pathway are manifestations of mitochondrial dysfunction and abnormal fatty acid metabolism in NAFLD.

In addition, PPARα might regulate the transcription of cytochrome P450 families (CYPs).^28^ Our study demonstrated that PPARα indirectly associates with cyp2j4 through cyp4a14 and induces subsequent metabolic pathways that are involved in the inflammatory response and liver injury. The cyp2j sub-families catalyze many reactions involved in arachidonic acid metabolism and then promote the inflammatory response in hepatic and extra-hepatic tissues.^29, 30^ The increase in hepatic glutathione is a marker of oxidative stress,^31^ and the increase in phosphatidylcholine and phosphatidylethanolamine indicates abnormal arachidonic acid metabolism and autophagic regulation, which may induce the inflammatory response in NAFLD. The decrease in hepatic choline damages the hepatocellular membrane integrity and might lead to liver injury with abnormal bile secretion.

The potential limitations of our study are that the specific regulatory relationships between genes have not been elucidated in our study, and whether changes in the mRNA levels of certain genes and concentration of metabolites reflect processes that are secondary to disease pathogenesis is also an open question. Thus, the causality between these genetic/metabolic changes and NAFLD progression needs to be determined by specifically designed studies, which would be more persuasive if functional experiments would support our claims going forward.

In summary, the current study demonstrates that ccl2 and jun are key regulators in all three NAFLD phenotypes and lead to abnormal fatty acid metabolism and inflammatory response through activation of TNF, P53, NF-κB, chemokine, PPAR and TLR signaling pathways. The specifically regulated genes and metabolites in HFD, MCDD and HFD+STZ rats are involved in lipid and fatty acid metabolism, immune and inflammatory response, and response to insulin, respectively. These results provide functional network-based regulatory models in the context of different NAFLD phenotypes with data derived from liver transcriptomic analysis and liver and serum metabolomics. These aggregated data complement the adaptive genetic and metabolic pathways that are involved in NAFLD and help us better understand the genetic and metabolic features in this disease with hepatic and extra-hepatic manifestations.

## Figures and Tables

**Table 1:** The body weight and biochemical indexes of rats in different groups

	*Group*			
*Parameters*	*Control*	*HFD*	*MCDD*	*HFD+STZ*
Body weight (g)	545.80±12.86	681.0±28.47*	313.70±9.22*	657.60±23.81*
ALT (U l^−1^)	42.30±2.53	147.90±32.75	422.90±63.58*	102.60±16.73
AST (U l^−1^)	107.70±3.50	190.30±22.48	587.90±66.00*	171.20±19.20
TBil (μmol l^−1^)	2.18±0.17	2.25±0.14	6.24±0.44*	1.89±0.11
TG (mmol l^−1^)	0.80±0.06	1.35±0.20*	1.70±0.24*	1.66±0.17*
HDL (mmol l^−1^)	0.37±0.02	0.40±0.01	0.35±0.05	0.37±0.03
LDL (mmol l^−1^)	0.13±0.01	0.38±0.20	0.78±0.17*	0.08±0.01
FBG (mmol l^−1^)	4.90±0.12	6.68±0.27*	4.37±0.17	13.65±0.99*
FINS (mIU l^−1^)	8.50±0.80	23.73±1.99*	7.06±0.62	17.66±1.62*
HOMA-IR	1.85±0.18	7.15±0.80*	1.35±0.11	11.29±1.43*

Abbreviations: ALT, alanine aminotransferase; AST, aspartate aminotransferase; FBG, fasting blood glucose; FINS, fasting insulin; HDL, high-density lipoprotein; HOMA-IR, homeostasis model assessment of insulin resistance; LDL, low-density lipoprotein; TBil, total bilirubin; TG, triglyceride.

Data are expressed as the means±s.e.m. *P<0.05 compared with control group.

**Table 2:** Pathway enrichment analysis for genes commonly changed

*Pathway*	*Genes*	P*-values*	*Database*
TNF signaling pathway	Ccl12, Icam1, Ccl2, Cxcl2	1.99E-04	KEGG
p53 signaling pathway	Cdkn1a, Serpine1, Rprm	4.75E-03	KEGG
NF-kB signaling pathway	Cxcl2, Icam1, Bcl2a1	1.41E-02	KEGG
Chemokine signaling pathway	Ccl2, Ccl3, Ccl12, Cxcl2, Cxcl10	1.65E-02	KEGG
PPAR signaling pathway	Fabp4, Fabp5, Olr1	6.27E-02	KEGG
TLR signaling pathway	Ccl3, Cxcl10, Jun	9.18E-02	KEGG

**Table 3:** Pathway enrichment analysis for metabolites commonly changed

*Pathway*	*Metabolics*	P*-values*	*Database*
Bile secretion	Vitamin D2 glucosiduronate, choline, glutathione, spermidine	6.34E-05	KEGG
Glutathione metabolism	Glutathione, spermidine	3.56E-03	KEGG
Arachidonic acid metabolism	Phosphatidylcholine	1.67E-02	KEGG
Unsaturated fatty acids biosynthesis	Clupanodonic acid	1.22E-01	KEGG
Mitochondrial beta-oxidation of long chain saturated fatty acids	Stearoylcarnitine	*	HMDB
Fatty acid degradation	Palmitoyl-L-carnitine	*	HMDB
Carnitine/acylcarnitine translocase	Elaidic carnitine	*	HMDB
Regulation of autophagy	Phosphatidylethanolamine	*	Pubmed
